# Toward a Systematic Structural and Functional Annotation of Solute Carriers Transporters—Example of the SLC6 and SLC7 Families

**DOI:** 10.3389/fphar.2020.01229

**Published:** 2020-08-19

**Authors:** Claire Colas

**Affiliations:** University of Vienna, Department of Pharmaceutical Chemistry, Vienna, Austria

**Keywords:** solute carriers, structural and functional annotation, drug discovery, LeuT-fold, structure-based ligand discovery

## Abstract

SLC transporters are emerging key drug targets. One important step for drug development is the profound understanding of the structural determinants defining the substrate selectivity of each transporter. Recently, the improvement of computational power and experimental methods such as X-ray and cryo-EM crystallography permitted to conduct structure-based studies on specific transporters having important pharmacological impact. However, a lot remains to be discovered regarding their dynamics, transport modulation and ligand recognition. A detailed functional characterization of transporters would provide opportunities to develop new compounds targeting these key drug targets. Here, we are giving an overview of two major human LeuT-fold families, SLC6 and SLC7, with an emphasis on the most relevant members of each family for drug development. We gather the most recent understanding on the structural determinants of selectivity within and across the two families. We then use this information to discuss the benefits of a more generalized structural and functional annotation of the LeuT fold and the implications of such mapping for drug discovery.

## Introduction

Solute carriers (SLC) transport a large variety of nutrients and metabolites across the cell membranes. Any dysregulation of these proteins function induces various disorders and diseases. For example, mutations in the Na^+^/citrate cotransporter NaCT (SLC13A5) lead to epilepsy and developmental delay (Klotz et al., 2016). Additionally, SLCs have an essential role in the absorption, distribution, metabolism, and elimination (ADME) of therapeutic drugs (Giacomini et al., 2010). For example, the peptide transporter PepT1 (SLC15A1) regulates the intestinal absorption of peptide-like drugs, such as β-lactam antibiotics (cefadroxil) and antiviral drugs (valacyclovir) across the cell membrane (Wenzel et al., 1995; Tamai et al., 1997). Thus, SLC transporters are crucial drug targets, where a drug can be a substrate binding an intracellular target or an inhibitor preventing the transport of endogenous substrates. Interestingly, in the past decade, five drugs targeting SLCs VMAT2 (SLC18A2), URAT1 (SLC22A12), SGLT2 (SLC5A2), and ASBT (SLC10A2) were approved by the Food and Drug Administration (FDA) and Japanese Pharmaceuticals and Medical Devices Agency (PMDA) (Garibsingh and Schlessinger, 2019).

Despite their pharmacological importance, SLCs have been understudied for many years (Cesar-Razquin et al., 2015). However recently, the awareness of the implication of SLCs in health has amplified and consequently, SLCs have been receiving increasing attention (Lin et al., 2015; Garibsingh and Schlessinger, 2019). For instance, the ReSOLUTE consortium is one of the actions taken to tackle the research on SLCs. This consortium is a public-private partnership created with the goal of improving knowledge of SLC transporters, demonstrating the importance of this field of research (Superti-Furga et al., 2020).

Molecular modeling has been proven to be a useful tool to conduct structure-based ligand discovery studies on SLC transporters (Colas et al., 2016; Schlessinger et al., 2018; Garibsingh and Schlessinger, 2019; Jiang et al., 2020). A key step toward increasing the success rate of drug discovery for solute carriers is a more comprehensive understanding of their substrate specificity and mechanism of transport. Thus, the description of the structural determinants defining the binding of ligands across the SLC family is essential to achieve. The recent characterization of several human structures of transporters and their homologs provided new opportunities to conduct structure-based studies (Gruswitz et al., 2010; Deng et al., 2014; Arakawa et al., 2015; Deng et al., 2015; Coleman et al., 2016a; Coleman et al., 2016b; Canul-Tec et al., 2017; Coleman and Gouaux, 2018; Garaeva et al., 2018; Coleman et al., 2019; Lee et al., 2019; Yan et al., 2019; Yan et al., 2020). Homology modeling is a recognized method employed to study a target lacking an available structure. However, this process requires a series of steps that need to be applied cautiously to ensure the accuracy of the final model (Colas et al., 2016; Schlessinger et al., 2018). Furthermore, several refinement steps can be performed. Particularly, the applicability of the model for ligand discovery using virtual screening can be evaluated with enrichment calculations, determining the ability of the model to prioritize ligands and decoys with docking.

Although very useful, homology modeling captures static conformations of the studied transporter. However, understanding how the transport cycle is achieved is of the utmost importance to design conformation specific modulators. In fact, a wide range of computational methods have been recently used to characterize the transport cycle at the molecular level (Jiang et al., 2020). For instance, molecular dynamics simulations are commonly used to explore the dynamics of proteins (Lindahl and Sansom, 2008). Furthermore, atomic structures of prokaryotic homologs and a few human transporters were released in various conformations of the transport cycle, which can be used as templates to model distinct conformational states (Colas et al., 2017).

Interestingly, the available structures reveal a significant variety of folds (Kuhlbrandt, 2014; Ceska et al., 2019). Among those, three main folds represent the majority of the transporters population to which specific transporter mechanisms have been associated (Colas et al., 2016; Drew and Boudker, 2016). In general the transport mechanism has been described as ‘alternating access’, where the transporter alternates conformations form outward-open to inward-open states to transport the substrates across the membrane. This alternating access mechanism is characterized by inverted structural motifs related by internal symmetry (Forrest et al., 2011).

The three main folds comprise: i) the LeuT fold, associated to the gated-pore mechanism of transport (also named rocking bundle). In this case one scaffold domain remains static while the mobile bundle domain opens and closes to capture and release the substrate. ii) Major facilitator superfamily (MFS), associated to the rocker switch mechanism. In this fold, the transporter is divided in two domains oscillating back and forth along an axis perpendicular to the membrane. iii) The Glt_Ph_-like fold, associated to the elevator mechanism. In this case, one mobile domain moves up and down across the membrane while the scaffold domain remains static.

In this review, we will focus on the first type, i.e., transporters presenting a LeuT-fold.

The prokaryotic leucine transporter LeuT shares 20–30% of sequence identity with the human neurotransmitter sodium symporters (NSS) and has been studied as a model transporter of the NSS superfamily (Beuming et al., 2006). The NSS comprise the serotonin, dopamine, and norepinephrine transporters (SERT, DAT, and NET, respectively) of the SLC6 family. Human NSS have been widely explored, due to their considerable pharmacological impact (Gether et al., 2006; Kristensen et al., 2011; Navratna and Gouaux, 2019). Furthermore, LeuT has been used as representative of all transporters of distinct families sharing a similar fold. In fact, the SLC5, SLC6, and SLC7 families revealed to be sharing this three-dimensional fold, as observed from the X-Ray structures of their closest prokaryotic homologs vSGLT, LeuT, and AdiC respectively. Furthermore, sequence alignments and clustering revealed that these families were evolutionary linked (Schlessinger et al., 2013), and were thus likely to use a similar transport mechanism, i.e., the gated pore mechanism.

Most drug discovery studies on SLCs have been focused on one specific transporter or a subgroup of transporters within a family. These studies have improved tremendously our understanding of how ligand recognition is achieved. Yet, the important question of how the conservation of a similar fold and transport mechanism allows substrate selectivity remains mostly unanswered. This review aims at shedding the light on the importance of collecting the most recent understanding on structure-function relationships of individual transporters to create a general knowledge on a specific fold and transport mechanism. We will focus on two human SLC transporters families sharing the LeuT-fold, i.e., SLC6 and SLC7, with an emphasis on the transporters with an important pharmacological impact.

### Two Major Human LeuT-Fold Families With High Pharmacological Impact

#### SLC6

The SLC6 family comprises neurotransmitters, amino acids, betaine, taurine, and creatine transporters. The first three subgroups contain transporters responsible for the synaptic reuptake of neurotransmitters essential in the regulation of neuronal communication. These transporters co-transport with their substrates 2 or 3 Na^+^, as well as 1 or 2 Cl^−^. Sequence similarity profiles and substrate specificities allow the division of the family into four subgroups (Kristensen et al., 2011).

- The monoamine transporter (MAT) subgroup that comprises the serotonin SERT (SLC6A4), dopamine DAT (SLC6A3) and norepinephrine NET (SLC6A2) transporters. The pharmacology of these transporters has been studied for many years, due to their high therapeutic impact (Gether et al., 2006; Kristensen et al., 2011; Navratna and Gouaux, 2019). Particularly the monoamine transporters have been targeted to treat depression, anxiety, and other neurological disorders. For instance, the selective serotonin reuptake inhibitors (SSRIs) such as escitalopram are specifically targeting the serotonin transporter SERT and administered in case of depression.
- The GABA transporters (GAT) subgroup. GABA is the main inhibitory neurotransmitter in the brain and is therefore a target for treating epilepsy. In humans, four distinct transporters are responsible for the transport of this neurotransmitter, namely GAT1 (SLC6A1), GAT3 (SLC6A11), BGT1 (SLC6A12), GAT2 (SLC6A13). This subgroup also comprises the transporters of the osmolyte taurine and betaine TauT (SLC6A6), as well as creatine CreaT (SLC6A8), which is a storage compound for high energy phosphate bonds.
- Amino acid (AA) I—including the glycine transporters. Glycine is another important inhibitory neurotransmitter of the central nervous system and is transported by two distinct transporters—GlyT1 (SLC6A9) and GlyT2 (SLC6A5), mainly located in the brain and the spinal cord, respectively. These transporters are targeted for the treatment of pain and schizophrenia. This group also contains two other amino acid transporters, the proline transporter PROT and neutral and amino acid transporter ATB^0,+^.
- Amino acid II. The fourth group contains amino acid transporters most probably responsible for amino acid homeostasis.

#### SLC7

The SLC7 family comprises cationic amino acid transporters (CATs) and L-type amino acid transporters (LATs) (Fotiadis et al., 2013). LATs are called light subunits and oligomerize with heavy subunits of the SLC3 family, i.e., 4F2hc (SLC3A2) or rBAT (SLC3A1), to form the heteromeric amino acid transporters (HATs).

LAT1 (SLC7A5) in particular has been subject of interest because of its pharmacological importance. Specifically, LAT1 is a neutral amino acid exchanger located in the blood brain barrier (BBB) and has thus been investigated to deliver drugs into the brain. Interestingly, L-DOPA (Kageyama et al., 2000; Soares-da-Silva and Serrao, 2004) and gabapentin (Wang and Welty, 1996; Dickens et al., 2013) are two examples of drugs crossing the BBB *via* LAT1. Thus, this transporter can be targeted by substrate compounds that can serve as prodrugs with optimal BBB permeability (Walker et al., 1994; Killian et al., 2007; Gynther et al., 2008; Peura et al., 2013; Rautio et al., 2013; Huttunen et al., 2016; Puris et al., 2017). Furthermore, LAT1 has been shown to be overexpressed in various cancers (Jin et al., 2015; Nagamori et al., 2016; Cormerais et al., 2016; Kongpracha et al., 2017). Thus, an inhibitor targeting LAT1 could act as an anti-cancer agent starving the cancer cell (Shennan and Thomson, 2008; Imai et al., 2010; Huttunen et al., 2016; Kongpracha et al., 2017). Consequently, LAT1 is a transporter of particular interest, as both drug substrates and inhibitors are needed. In fact, understanding the structural determinants discriminating substrates *vs.* inhibitors is one of the most challenging tasks when designing small molecules targeting transporters.

### Transport Mechanism

**Figure 1:**
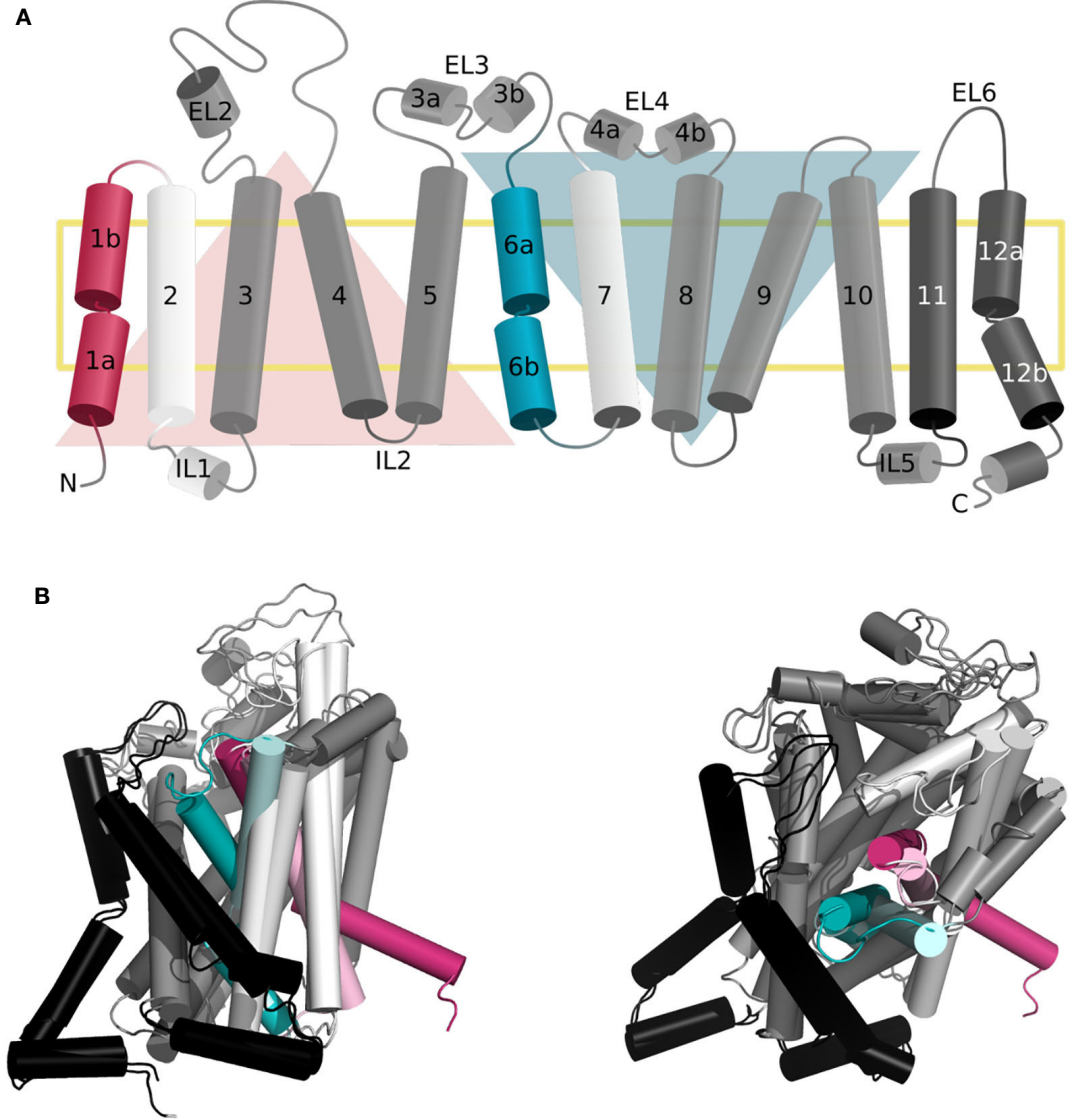
Topology of the gated pore mechanism of transport. **(A)** Topology of hSERT. The transmembrane helices constituting the bundle and scaffold domain are shown in white and dark gray, respectively, with the exception of the gating helices TM1 and TM6 in red and cyan. The two inverted repeats are indicated by two triangles, while the two additional helices TM11 and TM12 are shown in black. This figure was adapted from ref (Hellsberg et al., 2019). **(B, C)** Superposition of the three-dimensional structures of hSERT in outward open [PDB ID 6DZY (Coleman et al., 2019)] and inward open [PDB ID 6DZZ (Coleman et al., 2019)] conformations from the side **(B)** and top **(A)** view, with the same color code as panel **(A)** the gating helices in the outward open conformation are shown in light red and light cyan, to contrast with the inward open state.

As mentioned above, the LeuT-fold is one of the three main folds observed in SLC transporters, together with the MFS and elevator folds (Colas et al., 2016; Drew and Boudker, 2016). LeuT fold transporters are constituted of a two repeats core of five transmembrane segments (TMs 1–5 and TMs 6–10), related by a pseudosymmetrical axis parallel to the membrane (**Figure 1A**) (Forrest, 2015). The helices of each repeat arrange in two distinct domains, i.e., the scaffold (TM3-5 and TM8-10) that is more rigid and bundle domain (TM1,2 and TM6,7) that experiences conformational changes to capture and release the substrate (Abramson and Wright, 2009; Forrest and Rudnick, 2009).

The distinct crystal structures of LeuT (Yamashita et al., 2005; Krishnamurthy and Gouaux, 2012; Penmatsa and Gouaux, 2014; Malinauskaite et al., 2016; Gotfryd et al., 2020). as well as other homologs sharing a similar fold such as MhsT (Malinauskaite et al., 2014), BetP (Ressl et al., 2009; Perez et al., 2012), vSGLT (Faham et al., 2008; Watanabe et al., 2010), AdiC (Gao et al., 2009; Ilgu et al., 2016), and dDAT (Penmatsa et al., 2013) in various conformational states gave insight into the structural rearrangement necessary for transport to occur.

Recently, structures of human transporters SERT (Coleman et al., 2016a; Coleman et al., 2016b; Coleman and Gouaux, 2018; Coleman et al., 2019) and LAT1 (Lee et al., 2019; Yan et al., 2019). considerably enriched our understanding of the dynamics of transport. Particularly, the release of the hSERT structures in distinct conformational states (Coleman et al., 2016a; Coleman et al., 2016b; Coleman and Gouaux, 2018; Coleman et al., 2019) confirmed the predictions originally inferred from the LeuT structures (**Figure 1**). Moreover, the increasing number of human cryo-electron microscopy (EM) transporter structures complement those of the prokaryotic homologs and provide new opportunities for structure-based ligand discovery, for example when new conformations are revealed. Furthermore, these structures inform already available models of SLC transporters and allow new optimization steps. In fact, the comprehensive characterization of proteins using structure-based methods is a constant dialog between computational modeling and biological experiments, to build and refine transporter models as accurately as possible (Schlessinger et al., 2018; Jiang et al., 2020).

Importantly, these various structures also shed the light on some diversity occurring in this conserved fold.

#### Common Features

The dynamic of transport requires concerted global and local movements of the bundle domain. Specifically, the extracellular side is constituted of conserved gating residues i.e., (R30–Q404 in LeuT) and a hydrophobic lid (i.e., Y108 and F253 in LeuT). In fact, F253 is an aromatic gating residue described in various LeuT fold transporters that flips open simultaneously to a tilting of the extracellular regions of TM1b and 6a (**Figures 1B, C**) (Drew and Boudker, 2016; Navratna and Gouaux, 2019). The intracellular gate is characterized by the intracellular segment of TM1 (TM1a) tilting outward of the binding site to release the substrate (**Figures 1B, C**) (Drew and Boudker, 2016; Navratna and Gouaux, 2019). In fact, TM1 and 6 are characterized by unwound regions that have been shown to be of the outmost functional significance (Ponzoni et al., 2018). Specifically, a GXG motif has been identified in the TM1 of all available structures of transporters presenting a LeuT fold. This motif is suspected to provide the necessary flexibility for the opening and closing of the bundle domain and to transmit the conformational change of the extracellular to the intracellular side of the two helices.

#### Diversity

Interestingly, the different oligomerization states observed for LeuT fold transporters has been suspected to modulate the transport dynamics. Consequently, distinct helices involved in the oligomerization interfaces have restricted mobility from one transporter to the other (Ponzoni et al., 2018).

Furthermore, some variability has been observed in which transmembrane helix accomplishes the gating function. In fact, in the prokaryotic transporters BetP (Ressl et al., 2009; Perez et al., 2012) and vSGLT (Faham et al., 2008; Watanabe et al., 2010), TM5 and 10 act as gating helices from the cytoplasmic and extracellular sides, respectively.

Additionally, while the inverted repeat core of the LeuT fold is constituted of 10 transmembrane helices, there is a variability in the total number of helices from 11 (such as the prokaryotic tyrosine transporter Tyt1 (Quick et al., 2006)) to 14 (such as vSGLT (Faham et al., 2008; Watanabe et al., 2010)).

The number of co-transported ions is another variable feature. While the SLC6 family co-transports 2 to 3 Na^+^ ions, the LATs subgroup of the SLC7 family, to which LAT1 belongs are mostly exchangers.

This suggests that many factors contribute to the broad dynamic and functional plasticity within a conserved fold.

### Central Binding Site

#### SLC6

**Figure 2:**
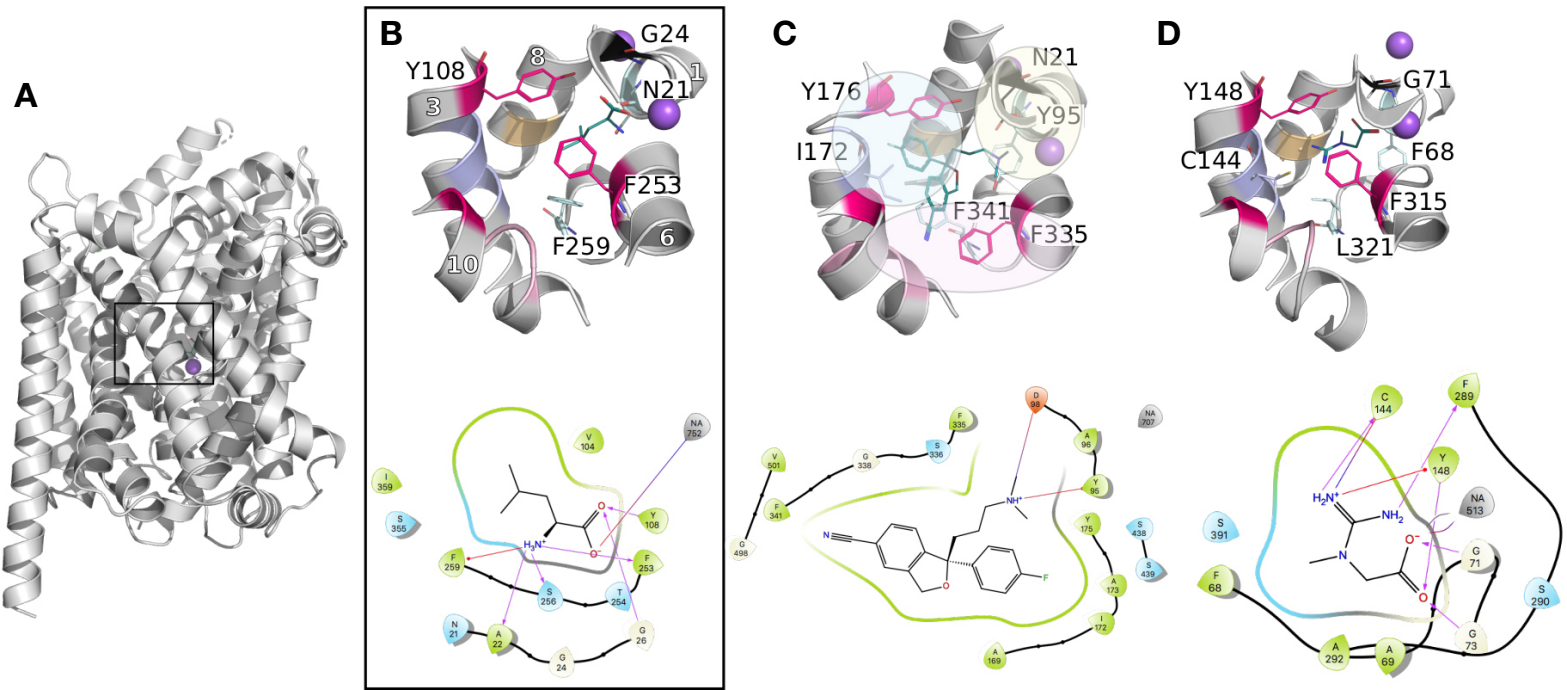
Conserved binding site organization of the SLC6 family. Binding sites comparisons between LeuT and representative human members of the MATs and GATs subgroups. The proteins are shown in gray cartoons with Na^+^ and Cl^−^ ions as purple and green spheres, respectively. The key amino acids constituting the binding site are labeled and shown in lines with the following color code: conserved gates are shown in pink; residues anchoring the ligands in black and cyan (TM1 and 6); amino acids involved in substrates specificities are shown in light pink, purple, and orange (TM10, 3, and 8 respectively). Finally, the bound ligands are shown in teal lines. For each complex, the 2D representation are shown in the bottom panel. The 3D representations have been generated with Pymol (Schrodinger), and the 2D with Maestro (Schrödinger, 2019). **(A)** Three-dimensional structure of LeuT (PDB ID 2A65 (Yamashita et al., 2005)) bound to Leucine. The black square locates the binding site. **(B)** A close up of LeuT binding site is shown, with the helices numbers indicated in white. **(C)** Binding site of the crystallographic structure of hSERT (PDB ID 5I73 (Coleman et al., 2016b)) bound to escitalopram. The subpockets A, B, C as reported in literature (Navratna and Gouaux, 2019; Cheng and Bahar, 2019) for the monoamine subgroup are represented with yellow, blue, and pink spheres, respectively. **(D)** Binding site of a homology model of the creatine transporter (Colas et al., 2020).

The primary binding site has been largely described for the prokaryotic transporter LeuT, the closest prokaryotic homolog of the SLC6 family (20–30% identity). This site is located in between transmembrane helices 1, 3, 6, 8, and 10 approximately halfway across the transmembrane bilayer (**Figure 2A**). The recent structures of the human serotonin transporter SERT (hSERT, SLC6A4) (Coleman et al., 2016b; Coleman and Gouaux, 2018; Coleman et al., 2019), as well as the drosophila DAT (dDAT) (Penmatsa et al., 2013). provided insight into the three-dimensional architecture of the monoamine transporter subgroup. Particularly, the binding site has been reported as constituted of three subpockets, A, B, and C (**Figure 2C**) (Navratna and Gouaux, 2019; Cheng and Bahar, 2019). Specifically, subpockets A (TM1b, 6, and 8) has been described as critical for the binding of the amine moiety of the substrates and is constituted of highly conserved residues (i.e., Q98 and Y95 in hSERT). Subpocket B (TM3 and 8) establishes hydrophobic interactions with the aromatic moieties of the ligands, and has been proposed to be involved in ligand specificity. Finally, subpocket C (TM3, 6A, and 10) shapes the binding site, and contains conserved aromatic residues (i.e., the gate F335 and F341 in hSERT).

The functional role of these subpockets can be expanded to the other subgroups of the SLC6 family. For instance, the corresponding area of the so-called subpocket A and C similarly has a ligand anchoring function. However, some variability takes place due to the different nature of the substrates between the subgroups. For example, the substrates of the GAT subgroup are constituted of a carboxylate moiety, interacting with a conserved glycine that substitutes the conserved aspartate within the monoamine subgroup (**Figure 2B**).

Furthermore, the reported influence of the subpocket B area in substrate specificity in the monoamine subgroup is also transferable to the other members of the SLC6 family. Specifically, the C144 in TM3 of the creatine transporter (CreaT, SLC6A8) has been shown to be deprotonated and suspected to be involved in ligand binding specificity (**Figure 2D**) (Drew and Boudker, 2016; Ceska et al., 2019). Interestingly, the other GATs carry a glycine at this position. However, this substitution is compensated by a cysteine (for the GABAs) or an aspartate (for TauT) at the same level in TM8, while in both positions, the monoamines carry hydrophobic residues (I172 and G442 in SERT).

Furthermore, multiple sequence alignment of the GAT subgroup with the rest of the SLC6 members revealed an additional residue in TM10. This multiple insertion has been discussed by our group and others as being a π-helix signature (Vogensen et al., 2015; Dayan et al., 2017; Kickinger et al., 2019). In particular, recent studies on several transporters from the GAT subgroup, including GAT1 (SLC6A1) (Dayan et al., 2017), hBGT1 (SLC6A12) (Jorgensen et al., 2017), and CreaT (Colas et al., 2020), included a refined homology modeling protocol to optimize this area of TM10 as a π-helix. This feature seems to be specific to the GAT subgroup of the SLC6 family. Additionally, our study on the CreaT revealed that an optimal ligand length of 4.5–5 Å seems necessary between the guanidine and carboxylate groups to establish hydrogen bonds with respectively C144 and G71 and the Na^+^.

Overall, several key features contribute to the various substrate selectivities of the SLC6 transporters. For instance, residue substitutions influence the physico-chemical properties of the binding site. Building on the functional annotation of the monoamine subgroup, a general trend can be drawn: i) conserved (within each subgroup) residues anchor the substrates in TM1 and 6, ii) while the residue variability occurring in TM3, 8, and 10 confers selectivity.

#### SLC7

In an effort to find compounds with optimal affinity/transport properties for LAT1, integrative multidisciplinary studies have been conducted, comprising computational modeling and experiments (Chien et al., 2018). The recent release of cryo-EM structures (Lee et al., 2019; Yan et al., 2019) of the LAT1-4F2hc complex provided precious insight into the architecture of the dimer. Furthermore in these structures, LAT1 presents an inward open state, complementing the previous studies relying on homology models based on the closest prokaryotic homolog AdiC in outward open and outward occluded conformations (Gao et al., 2009; Ilgu et al., 2016).

**Figure 3:**
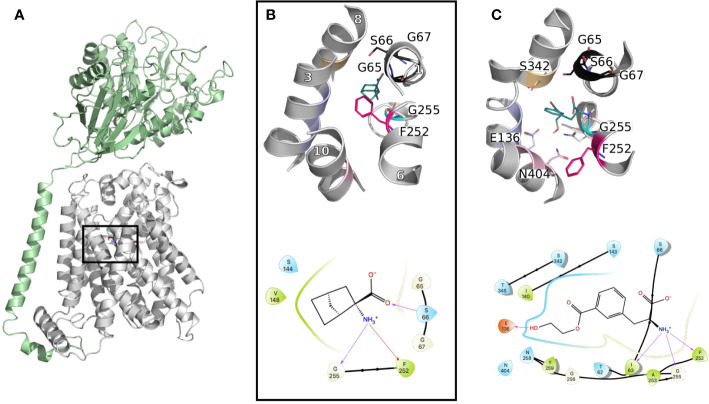
LAT1 binding site. **(A)** The cryo-electron microscopy (EM) structure of the LAT1-4F2hc complex (PDB ID 6irt (Yan et al., 2019)) is represented in cartoon. 4F2hc is shown in green and LAT1 bound to 2-amino-2-norbornanecarboxylic acid in gray, from a side view, parallel to the membrane plane. **(B)** Close up representation of the binding site from a top view. Important residues defining the binding site are labeled and shown in lines with the same color code as in **Figure 1**, and the helices are labeled in white. **(C)** Binding pose of a new reported potent substrate resulting from a study on a homology model of LAT1 (Chien et al., 2018). The bottom panels **(B, C)** show the two-dimensional representation of the LAT1 interaction with its ligands.

These data revealed the structural motifs characterizing the LeuT fold, such as a conserved aromatic gate (i.e., F254 in LAT1 *vs.* W202 in AdiC) and broken helices facilitating transport (**Figures 3A, B**).

Furthermore, comparisons of the binding sites of LAT1 and its prokaryotic homolog AdiC revealed conserved binding site residues interacting with the backbone of the transported amino acid. Particularly, the carboxyl moiety establishes interactions with the backbone of the conserved GXG motif of TM1, i.e., G65, S66, and G67, while the amino group interacts with G255 of TM6 (**Figure 3B**). Conversely, the residues interacting with the side chain of the transported amino acid are more variable between LAT1 and AdiC. This variability explains partly the substrate specificities of these two transporters. In fact, the residues in this area of the binding site are short and hydrophobic to accommodate the large neutral amino acids transported by LAT1. Conversely, AdiC is an arginine/agmatine exchanger and these positions are substituted by polar residues. Consequently, the LAT1 binding site is larger and hydrophobic and provides opportunities for the design of specific compounds. In fact, initial LAT1 models guided the development of new compounds, including tyrosine and phenylalanine derivatives (Augustyn et al., 2016), carboxylic acid bioisosteres (Zur et al., 2016), and other unique scaffolds. Particularly, a multidisciplinary study combining computational modeling using homology modeling and ligand docking, followed by experimental testing using synthetic chemistry and cellular uptake measurements, permitted the discovery of a potent substrate with an IC_50_ of 29 μM (**Figure 3C**) (Chien et al., 2018). Notably, one explanation of the reported substrate activity of this compound is the additional hydrogen bond with E136 (TM3) (**Figure 3C**). The docking pose also exhibits additional hydrogen bonds with S342 (TM8) and N404 (TM10) (**Figure 3C**). Altogether, these data show that LAT1 ligands are anchored by conserved residues in TM1 and 6, while their differential activities reside in their distinct interactions in TM2, 8, and 10.

**Figure 4:**
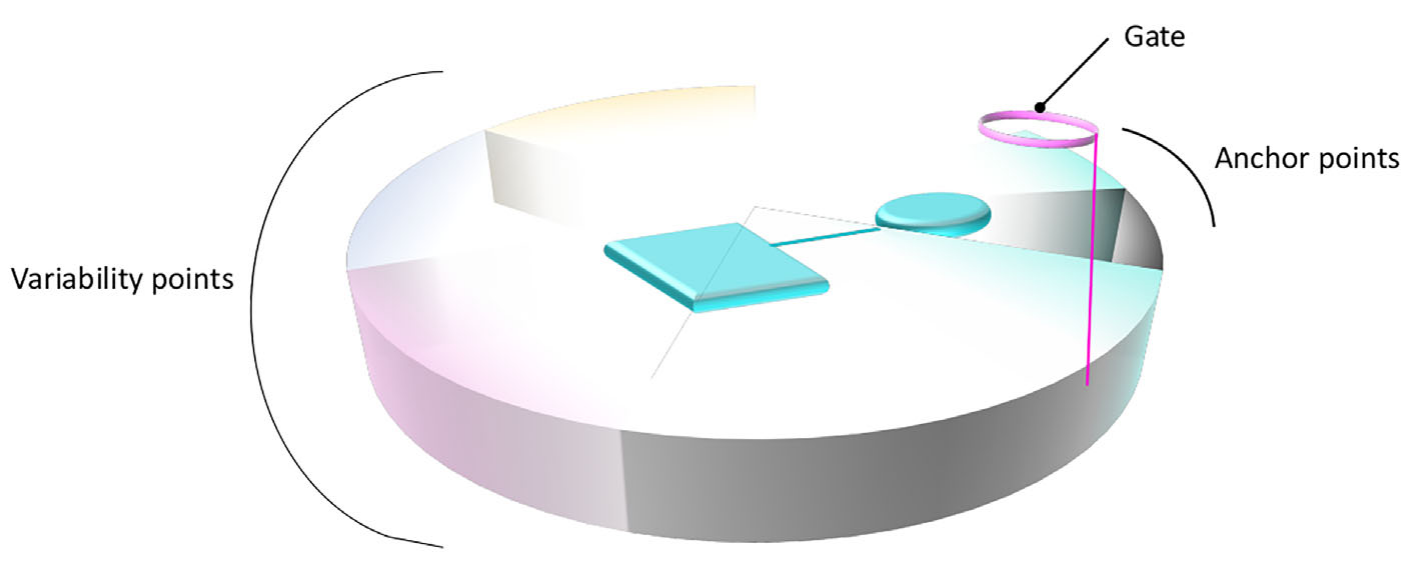
General structural annotation of the primary binding site of LeuT fold transporters. Gathering the information available from the distinct studies on the SLC6 and SLC7 members permits to structurally and functionally map the binding site of LeuT fold transporters. The binding site is symbolized as a disc divided into distinct portions that constitute the binding site, following the same color code as in **Figure 1**. The functional annotation of each area is indicated on the side of the disc. A bound ligand is represented in cyan. The anchored part of the ligand, conserved within subgroups is shown as a cylinder. The part of the ligand allowing variability and conferring various transport activities is shown as a square.

To summarize, the common binding patterns observed for distinct members of the SLC6 and SLC7 families provide evidence of a conserved binding site organization within the two families. Overall, the physico-chemical properties of the primary binding sites complemented with those of the substrates combine to confer the unique substrate specificity determinants of each transporter. This can lead to a generalized functional annotation of the primary binding site, as suggested in **Figure 4**.

### Toward a Global Structural and Functional Annotation of the LeuT-Fold

The previous sections demonstrate that the three-dimensional LeuT fold contains common patterns to achieve its transport function, while various local differences allow the diverse substrate specificities of each transporter. Members of proteins superfamillies are under evolutionary constrains to keep the balance between maintaining the three-dimensional architecture and its intrinsic dynamic, while sustaining amino acid substitutions to confer functional promiscuity within the superfamily.

**Figure 5:**
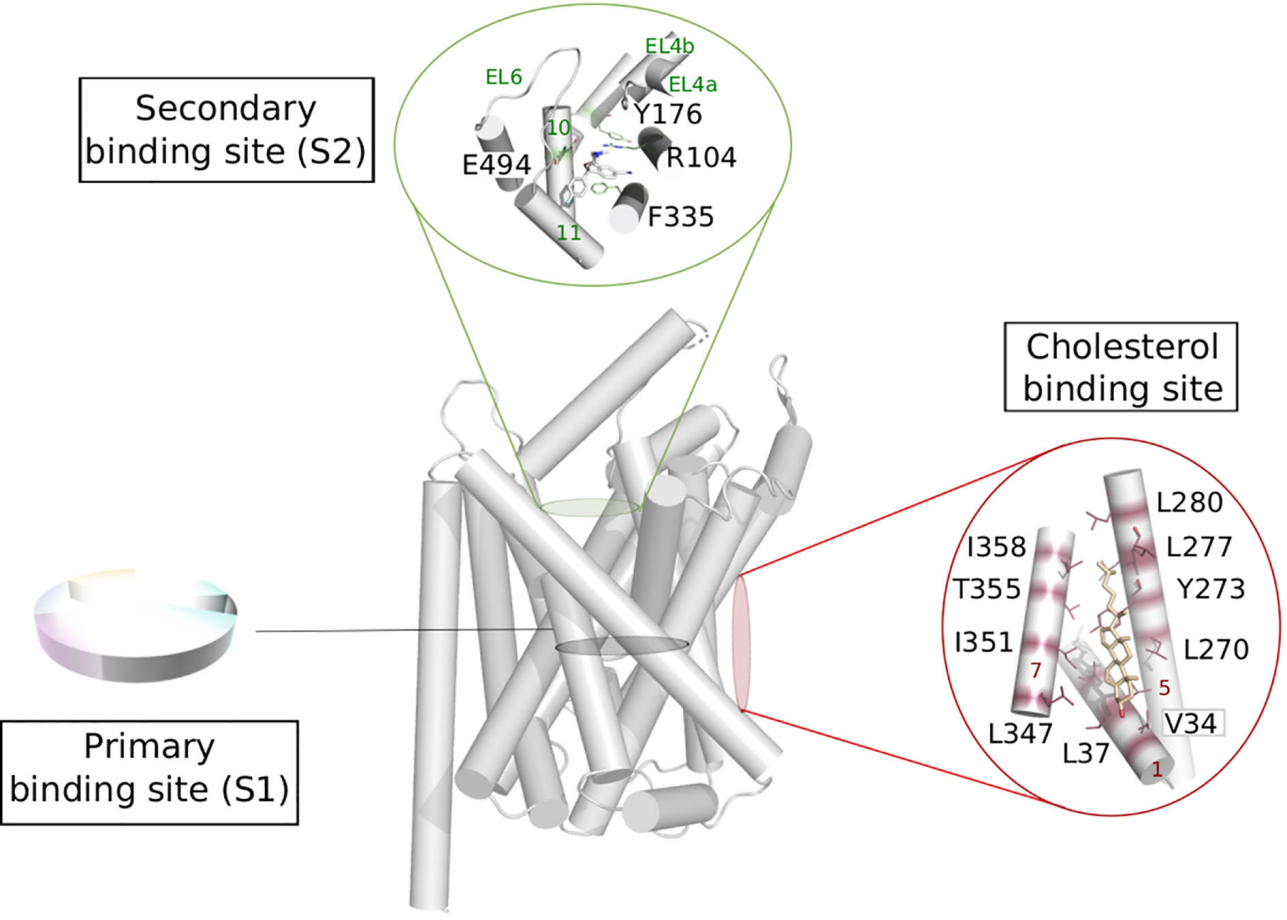
Possible functional mapping of the LeuT fold. Building on the functional mapping of the binding site proposed in **Figure 4**, we show here two additional area where functional mapping could be beneficial, i.e., on the secondary and cholesterol binding sites. The residues involved in binding are shown in sticks and labeled (hSERT numbering for the secondary site and dDAT numbering for the cholesterol binding site), the helices and loops are named on green and red on the secondary et cholesterol binding site, respectively. Expanded to the whole fold, a systematic annotation could help the identification of new sites that could be targeted in ligand discovery.

A general functional annotation of LeuT fold transporters could help highlighting the importance of new binding sites and guide the design of new compounds with novel scaffolds and new selectivity profiles. Such global mapping would be relevant for instance in the following areas of the transport process (**Figure 5**).

#### Localizing Substrate Specificity

The detailed descriptions of the interactions of distinct members of each family with their substrates in the primary binding sites revealed conservation of important functional positions serving as anchor points (on TM1 and 6), while integrating variability of residues at key positions allowing specific protein-ligand interactions (on TM3, 8, and 10) (**Figure 4**).

Interestingly, questions have been recently raised over where specificity is located in LeuT fold transporters. This has been particularly studied across the three monoamines transporters SERT, DAT, and NET, given their tremendous pharmacological implications. In fact, substrate specificity overlaps within these three transporters. For instance, SERT has been shown to transport dopamine at low affinity (Broer and Gether, 2012).

Deciphering precisely the structural determinants underlying the substrate specificities would provide opportunities to design specific modulators and decrease side effects.

Interestingly, mutational studies on the three human monoamines transporters suggested that specificity determinants might be more than non-conserved key residues in their respective binding site (Andersen et al., 2011; Andersen et al., 2015). Specifically, swapping residues constituting the primary binding sites between SERT and NET permitted to reverse the inhibitor stereo-selectivity preferences between the two transporters (Andersen et al., 2011). Similarly, inserting DAT residues in NET decrease the potency of NET inhibitors. However, surprisingly, introducing NET residues in DAT did not result in a NET-like selectivity profile of DAT, except for rimcazole (Andersen et al., 2015). This suggests that the determinants for selectivity within the three monoamine transporters are not exclusively due to non-conserved residues. Notably, transport kinetics is also an important aspect of selectivity. Particularly, a study showed that the association rate constant k_on_ is a determinant factor in the selectivity of specific inhibitors toward SERT and DAT (Hasenhuetl et al., 2015). This suggests that the entry pathway of ligands to the binding site also influences the affinity of ligands.

#### Secondary Binding Site

The extracellular vestibule of the LeuT fold is likely to be part of the entry pathway for the substrates toward the orthosteric site, also called S1. This area is located approximately 10 Å above the primary binding site and is enclosed by the TMs 1b, 6a, 10, 11 and EL2, 4, and 6 (**Figure 5**). However, the X-ray structures of LeuT and hSERT revealed a secondary site (also referred to as S2) located in this extracellular vestibule of the transporter, where serotonin uptake inhibitors (SSRIs) could bind (Zhou et al., 2009; Coleman et al., 2016b; Coleman and Gouaux, 2018). The functional relevance of this site has been subject of controversy over the years (Reyes and Tavoulari, 2011).

Computational calculations suggested that such site could be a transient spot along the transport pathway of the substrate (Celik et al., 2008; Grouleff et al., 2017). In a study addressing the substrate transport in GlyT2, the extracellular vestibule is suggested to act as a funnel directing the pool of substrates toward the primary site (Carland et al., 2018). However, the binding of a substrate in S2 in LeuT as well as in DAT was reported to trigger the opening of the intracellular gate, and thus, substrate release from the primary site, suggesting an allosteric function of S2 (Shi et al., 2008; Shan et al., 2011).

The extracellular loops EL4 and EL6 can adopt various conformations and thus contribute to partially shape the S2 binding site, indicating considerable plasticity of this secondary site, likely to accommodate small substrates or bulkier ligands. The latter category could be used as new inhibitors targeting this secondary site preventing the conformational change toward an inward facing state (Navratna and Gouaux, 2019).

Thus, a functional annotation of this site could guide the design of such new modulators. In fact, a very recent study already revealed the first hSERT high affinity S2 inhibitor, demonstrating the relevance of targeting such site in drug discovery (Plenge et al., 2020).

#### Cholesterol Modulation

Crystal structures of dDAT revealed two lipid binding sites namely site 1 (located at the interface of TM1a, TM5, and TM7 bound to cholesterol, **Figure 5**) and site 2 (found at the interface between TM2 and TM7, bound do cholesteryl hemisuccinate) (Penmatsa et al., 2013; Penmatsa et al., 2015; Wang et al., 2015). MD simulations on a homology model of hDAT suggested that cholesterol in site 1 stabilizes the outward open conformation of hDAT, by preventing the necessary tilt of TM1a and TM5 to occur for a conformational change toward an inward open conformation (Zeppelin et al., 2018). Due to the conservation of amino acids constituting this site within the monoamine transporters, the authors suggest that the stabilizing effect of cholesterol in site 1 can be expected in all monoamine transporters. This hypothesis has been confirmed in an adjacent study by the same group on hSERT, revealing how the cholesterol-modulated conformational change of the transporter relates to the kinetics variations of the transported substrates (Laursen et al., 2018). Specifically, the authors show that cholesterol binding in site 1 stabilizes the outward open conformation and increases serotonin uptake, while cholesterol depletion shifts the conformational equilibrium toward a more inward open state, and thus prevents serotonin uptake.

Assuming that transporters sharing the same fold use a similar mechanism of transport, it is reasonable to expect a similar lipid modulation for all LeuT fold transporters. In fact, a study showed that a depletion of cholesterol induced a reduction of uptake activity in LAT1 and predicts to share the cholesterol binding sites with dDAT (Dickens et al., 2017). This is an additional evidence that common mechanisms are at play to modulate gated-pore transport.

## Conclusions and Future Directions for Drug Discovery

The recent interest on SLC transporters led to a large number of research studies improving further our understanding on how membrane transport is achieved. Here, we discuss the benefits of structural and functional mapping of SLC transporters presenting a LeuT fold for structure-based ligand discovery, using as example the SLC6 and SLC7 families. We first gave an overview of the LeuT fold architecture and transport mechanism, and introduced two major families presenting this fold, i.e., SLC6 and SLC7. We then presented the current knowledge regarding the structural determinants of binding in the primary binding site of pharmacology relevant members of each family and showed that a general functional annotation encompassing both families is relevant. Finally, we discuss how the extensive mapping already well established for the primary site could be generalized and expanded to other sites for all transporters sharing the same fold, giving as example the cholesterol and secondary binding sites.

Allosteric modulation of SLC transporters and its pharmacological relevance is getting increased attention (Niello et al., 2020). Allostery has been extensively studied for several decades for many biological complexes, notably for ion channels and G protein-coupled receptors (GPCRs). Yet, very little is known regarding allosteric regulation of SLC transporters and has only been demonstrated on isolated cases. The development of new computational methods allowing the detection of secondary sites in an automated way would tremendously improve rational ligand discovery against these sites.

Furthermore, integrating the dynamic of transport in the drug discovery process remains a challenge, and is yet necessary to design conformation specific modulators. The recent improvement of cryo-EM methodologies (Kuhlbrandt, 2014; Ceska et al., 2019) permitted the release of several human transporters’ structures, complementing the available structures of homologs and allowing to progressively fill the conformational landscape of SLC transporters. These structures permitted to characterize key features such as the polarity, protonation, and shape of the binding site, which can influence greatly the differential binding and transport activities of ligands.

As most studies focus on individual transporters, detailed description of structure activity relationship studies of each SLC are becoming available. Gathering all this information in an integrative manner is a key step toward an holistic understanding of their function, and a more systematic and efficient way to specifically target each transporter (Rout and Sali, 2019).

Finally, such functional annotation would also be relevant for the two other main folds, i.e., the MFS and the Glt_Ph_-like fold, to ultimately further characterize each SLC transporters family and improve the design of new specific drugs targeting these key pharmacological proteins.

## Author Contributions

The author confirms being the sole contributor of this work and has approved it for publication.

## Funding

This work acknowledges funding from the Innovative Medicines Initiative 2 Joint Undertaking under grant agreement No 777372 (“RESOLUTE”). This Joint Undertaking receives support from the European Union’s Horizon 2020 research and innovation program and EFPIA.

## Conflict of Interest

The author declares that the research was conducted in the absence of any commercial or financial relationships that could be construed as a potential conflict of interest.
